# Real-Time Sign Language Interpretation via Customized Sign Language Gloves and Motion Retrieval

**DOI:** 10.3390/s26154884

**Published:** 2026-08-03

**Authors:** Chien-Hua Chen, Chih-Yuan Yao, Shih-Hsuan Hung

**Affiliations:** 1 Department of Computer Science and Information Engineering, National Taiwan University of Science and Technology, Taipei 106335, Taiwan; d10815002@mail.ntust.edu.tw (C.-H.C.); cy.yao@mail.ntust.edu.tw (C.-Y.Y.); 2 Department of Computer Science, National Tsing Hua University, Hsinchu 300044, Taiwan

**Keywords:** data glove, sign language interpretation system, motion recognition

## Abstract

A sign language interpretation system aims to translate sign gestures into spoken or written language in real time, enabling signers and non-signers to communicate in their familiar linguistic forms. However, vision-based approaches suffer from hand occlusion, lighting variability, and complex backgrounds, while Deep Neural Network (DNN)-based methods incur heavy computational costs that hinder real-time use on resource-constrained platforms. In this paper, we propose sign language gloves and a lightweight motion retrieval method for real-time sign language interpretation that runs on mobile devices and embedded systems. The sign language gloves integrate flex sensors, an inertial measurement unit (IMU), and pressure sensors to accurately capture gesture features, including finger bending angles, hand orientation, movement trajectories, and fingertip contacts with body parts, enabling recognition of touch-based gestures. For the motion retrieval method, we build a comprehensive gesture dataset with the gloves and perform feature analysis on each sign language gesture to avoid redundant information in the dataset. During interpretation, our system employs a feature-labeling mechanism to ensure gesture distinguishability and a gesture retrieval algorithm to evaluate movement continuity and similarity. This allows the system to identify corresponding feature labels and consolidate them into complete sign language vocabulary entries. The proposed motion retrieval method is characterized by low computational complexity and a well-defined data structure. This makes it suitable for integration into embedded systems, offering real-time performance and high portability for practical deployment. In our experiments, the proposed system achieved an average recognition accuracy of 92% on a gesture dataset covering 300 sign language words.

## 1. Introduction

Sign language is a vital means of communication for individuals with hearing or speech impairments. When people communicate, they often rely not just on spoken words, but also on tone, facial expressions, and body language. For those unfamiliar with sign language, this can lead to significant communication barriers. While tools like pen and paper, hearing aids, and digital messaging offer partial support, they often come with limitations. In response to growing public and governmental attention to disability services, sign language interpreters have become important facilitators. However, professional certification demands years of training and experience, and many users either lack access to such services or depend on family members, causing further delays. A mobile sign language translation system could offer a more immediate and accessible solution to bridge this communication gap.

Recent advances in sign language recognition have primarily focused on improving gesture capture and interpretation techniques. Since sign language consists of continuous hand gestures, the task is commonly formulated as a sequential gesture recognition problem. Existing approaches can be broadly categorized into two main types: vision-based methods [1,2,3,4,5,6,7] and data glove-based methods [8,9,10,11,12,13,14]. Vision-based methods often suffer from visual challenges such as occlusion and background interference, while data glove-based approaches tend to be expensive or may lack sufficient sensor coverage to accurately capture hand movements in sign language. Meanwhile, recent sign language recognition techniques—including deep learning models such as Long Short-Term Memory (LSTM) networks [14] and template matching methods such as Dynamic Time Warping (DTW) [10,13]—have achieved success in recognizing sign language. However, deep learning-based approaches often require extensive training data and computational resources, posing challenges for deployment on mobile and resource-constrained devices.

To address these challenges, we present a real-time and scalable sign language interpretation system designed for practical use in resource-limited environments. We develop a custom sign language glove that captures not only finger bending and hand orientation but also fingertip pressure, introducing touch as an additional modality for gesture representation. We then employ a lightweight motion retrieval algorithm based on *k*-nearest neighbors (*k*-NN) [15] and the Online Lazy Neighborhood Graph (OLNG) [16], enabling fast and scalable recognition through similarity-based matching while achieving consistent accuracy across the evaluated sign-language sets. This design makes our system particularly suitable for real-time deployment on resource-constrained devices.

To summarize, our primary contributions are as follows:

- Multi-modal sensor glove design: We design and integrate a sign language glove that combines flex sensors, a nine-axis inertial measurement unit (IMU), and fingertip pressure sensors. The inclusion of pressure sensing enables the system to distinguish gestures that involve finger–body contact, a common feature in sign language that prior glove designs often overlook.
- Lightweight end-to-end interpretation pipeline: We develop a complete recognition pipeline—from feature extraction and labeling to OLNG-based motion retrieval and voting-based result fusion—that runs in under 7 ms on a low-power mobile processor. Unlike deep learning approaches that require large training datasets, our system needs only a single recorded sample per gesture, making it practical for rapid vocabulary expansion and embedded deployment.

## 2. Related Work

This section reviews related work on sign language recognition, covering both gesture capture techniques and recognition algorithms. We first discuss the hardware approaches used to acquire gesture data, then examine the algorithmic methods employed for sign language recognition.

### 2.1. Techniques for Gesture Capture

Sign language consists of continuous hand gestures that convey lexical meanings. Therefore, this work treats sign language as a sequence of discrete gesture segments. In gesture capture research, considering the real-time performance and portability required for sign language translation, devices that are bulky or require complex setup environments are excluded. Based on the hardware used, the approaches are mainly categorized into two types: vision-based analysis and wearable devices.

In the scope of vision-based analysis, Ohn-Bar et al. [1] used color and depth images with Support Vector Machine (SVM) for hand segmentation and gesture classification. Oikonomidis et al. [4,5] employed Particle Swarm Optimization (PSO) to fit 3D hand models to depth images, achieving real-time tracking at 20 Hz on graphics processing unit (GPU)-powered devices. Deep learning approaches have also been explored, with methods using Convolutional Neural Networks (CNN) for hand pose estimation [6].

For wearable devices, Zhang et al. [8] used a data glove combined with Kinect [17] to capture hand gestures and body movements for interactive applications. Recent wearable approaches have explored diverse sensor configurations for sign language recognition. Lee et al. [9] and Chu et al. [13] demonstrated that integrating pressure or force sensors with flex sensors significantly improves recognition of gestures involving finger–body contact. Jani et al. [10] and Zhang et al. [14] achieved high accuracy using flex and IMU sensors through advanced algorithmic approaches. Achenbach et al. [11] evaluated data gloves for static hand-shape recognition using resistive bend sensors and reported strong classification performance with machine learning models. Ji et al. [12] combined flex sensors with inertial measurement units in a data glove system for sign language recognition, further demonstrating the potential of multi-sensor fusion in wearable approaches.

### 2.2. Algorithms for Sign Language Recognition

In sign language recognition, traditional approaches have employed Artificial Neural Networks (ANNs) and Hidden Markov Models (HMMs). Yang et al. [2] implemented the Conditional Random Fields (CRF) method with Adaptive Threshold to segment continuous gestures, achieving a recognition rate of 93.5% on 480 American Sign Language (ASL) gestures. Lee et al. [18] used Kinect [17] combined with HMM and SVM for gesture capture and classification, achieving a recognition rate of 85.14% on 25 Taiwanese Sign Language (TSL) gestures. Beyond HMM-based approaches, template matching methods such as DTW have been adopted for training-free recognition [10,13]. Recent studies have also explored deep learning models: Zhang et al. [14] achieved 98.63% accuracy on ASL alphabets using a customized LSTM network with K-Fold cross-validation. However, these data-driven models require substantial training data and computational resources, limiting their deployment on embedded devices.

Compared with training-intensive approaches, Tautges et al. [16] proposed motion similarity retrieval using accelerometers and the OLNG method. Inspired by their lightweight retrieval approach, we adopt a *k*-NN-based method with feature labeling to achieve real-time performance on resource-constrained platforms. Our system applies the OLNG concept with energy-based segmentation to retrieve the most similar gesture from the dataset.

In summary, existing glove-based systems either rely on computationally expensive deep learning models or lack sufficient sensor coverage (e.g., omitting touch sensing). Our work addresses both gaps by combining a multi-modal sensor glove—including fingertip pressure sensors for touch detection—with a lightweight retrieval-based recognition pipeline that requires no model training and runs in real time on embedded hardware.

## 3. Sign Language Gloves

As mentioned in [19], gestures can be subdivided into the following components:

1. Hand shape: When making gestures, various hand shapes can be formed according to the degree of bending of the five fingers of the hand.
2. Location: The spatial position where the gesture is performed relative to the body; different locations can distinguish signs that share the same hand shape.
3. Movement: The direction and speed of hand motion during gesture execution.
4. Orientation: The direction in which the hand is facing or pointing when making signs.

Therefore, compared to image analysis methods that are easily affected by environmental lighting, wearable devices can sense hand movements directly and accurately. To effectively capture gesture information, the system uses data gloves, installing flex sensors on the fingers and an IMU module on the back of the hand to directly sense hand movements. The data is then transmitted wirelessly to the terminal device for further algorithm processing.

**Figure 1:**
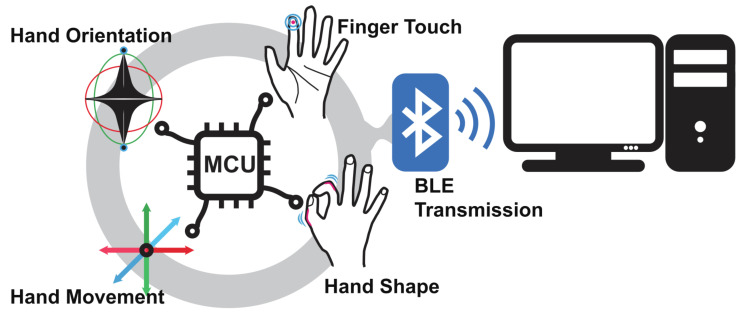
Hardware architecture diagram, illustrating the end-to-end data flow from sensor capture to wireless transmission. Flex sensors capture finger bending angles, pressure sensors detect fingertip contact, and a nine-axis IMU provides hand orientation and movement data. The microcontroller unit (MCU) on the main control board transmits all sensor readings to the terminal device via Bluetooth Low Energy (BLE).

In designing the sign language glove, we define four gesture features aligned with the structural components listed above: finger bending angle, hand orientation, hand movement, and touch. Each feature type is captured by a complementary sensor modality: flex sensors for finger bending (hand shape), an IMU for orientation and movement, and pressure sensors for touch. This multi-sensor design ensures that the system can distinguish gestures that differ in only one component (e.g., identical hand shapes with different contact patterns). As shown in Figure 1, bending sensors are used to capture the bending angles of the five fingers, including the joints between the palm and fingers and between the fingers. The hand orientation and movement are obtained through a nine-axis IMU, comprising a three-axis gyroscope, a three-axis accelerometer, and a three-axis magnetometer, to compute the orientation and movement direction. Finally, finger touch is detected by placing pressure sensors on the fingertips to sense the pressure applied. After collecting the above data, it is transmitted to the terminal device via Bluetooth Low Energy (BLE) and visualized using a three-dimensional hand model.

**Figure 2:**
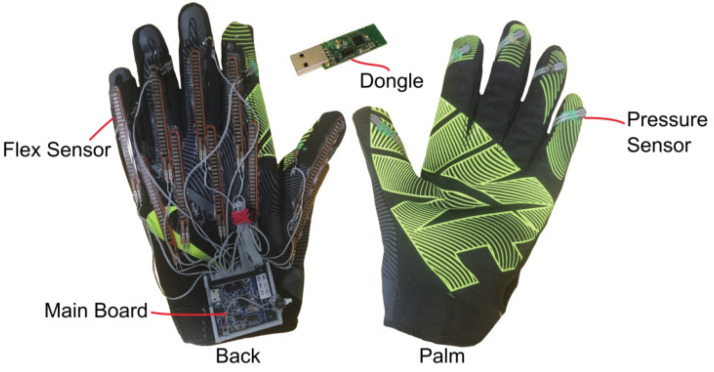
Assembled sign language glove showing sensor placement. The main control board is mounted on the back of the hand, bending sensors are aligned with the finger joints, and pressure sensors are attached to the fingertips for touch detection.

Finally, in the integration of sign language gloves, since we use palm and finger information as the gesture feature, the main control board is installed on the back of the hand to sense the overall rotation and movement of the palm. The bending sensors are attached to the metacarpophalangeal and interphalangeal joints to sense the bending state of the fingers, and the pressure sensors are sewn on the finger pads to detect finger contact, completing the assembly of the sign language glove. The completed sign language glove is shown in Figure 2.

## 4. Sign Language Interpretation

After designing and assembling the gloves, the first step is to convert raw sensor readings into four feature categories: finger bending, touch, hand orientation, and movement. All acquisition, calibration, and filtering are performed on the microcontroller of the main control board before transmission. The flex sensors and fingertip pressure sensors are sampled through an analog-to-digital converter (ADC), with the flex readings calibrated into finger bending angles and the pressure readings into fingertip pressure values. The nine-axis IMU is read over an inter-integrated circuit (I2C) bus: the gyroscope and accelerometer undergo zero-point calibration, and the magnetometer is corrected for hard-iron distortion. A Kalman filter [20] suppresses noise in the sensor signals, and the denoised inertial readings are fused by a Mahony filter [21] to estimate hand orientation as quaternions, while hand movement is represented as three-dimensional acceleration vectors. The processed features are transmitted to the terminal device over the BLE link at a 30 Hz sampling rate.

The second stage is the establishment of a sign language gesture dataset. When wearing the sensor gloves and making sign language gestures, the gesture features are recorded in real time via a wireless transmission protocol to establish the sign language dataset. In addition to encoding the dataset using a *k*-d tree, similarity calculations are performed on the feature data to establish labels and avoid duplicate data input, thereby speeding up the retrieval process.

The third stage is the actual sign language recognition process. After the user wears the sensor gloves, they can start making sign language gestures. The system searches each feature index for nearby samples using the *k*-NN algorithm. Individual OLNGs are then constructed to retrieve the labels represented by similar consecutive movements, and the label with the highest score is selected as the final result through a voting process. Finally, the corresponding sign language vocabulary is presented in text or voice.

### 4.1. Feature Extraction

**Figure 3:**
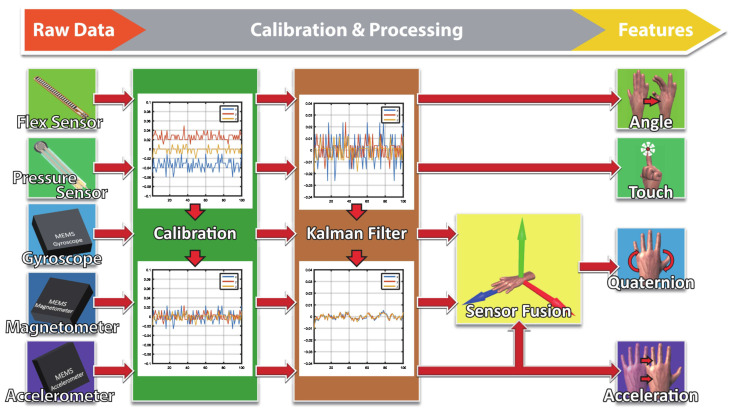
Feature extraction pipeline. Raw sensor readings from the glove are processed by the on-board microcontroller into four feature types before wireless transmission to the terminal device: finger bending angle (Angle, Θ) from the flex sensors, fingertip touch (Touch, *P*) from the pressure sensors, and hand orientation (Quaternion, *Q*) and hand movement (Acceleration, *A*) from the nine-axis IMU, which integrates the three-axis gyroscope, accelerometer, and magnetometer.

By selecting the desired gesture features and their corresponding hardware components, the next step is to obtain meaningful data from the sensors, as shown in Figure 3. Since the sign language glove utilizes a wireless transmission protocol, to avoid delays or packet loss during transmission, the collection of raw data, as well as the subsequent correction and filtering of the data, are all performed by the microcontroller on the main control board. The processed data is then transmitted to the terminal device to ensure the stability of the overall data.

After obtaining gesture data from the sign language glove, this system builds a sign language dataset to store the gestures recorded from the user. The sign language dataset contains *M* sign vocabulary, denoted as $S=\{s_{1},\ldots ,s_{M}\}$. Each sign vocabulary $s_{m}$ is composed of a sequence of continuous gesture motion frames, consisting of *N* sampled frames, forming a continuous action sequence $F=\{f_{1},\ldots ,f_{N}\}$. Each frame $f_{t}$ is composed of a set of gesture features $\left(\Theta_{l},P_{l},Q_{l},A_{l},\Theta_{r},P_{r},Q_{r},A_{r}\right)$ for the left (*l*) and right (*r*) hands, where Θ denotes finger bending angles, *P* denotes fingertip touch detection, *Q* denotes hand orientation, and *A* denotes hand movement acceleration.

### 4.2. Preprocessing

To enable fast retrieval in the gesture dataset, we encode each gesture frame using *k*-d trees [22], which support efficient nearest-neighbor search in multi-dimensional spaces. Each gesture frame comprises 44-dimensional feature values. Rather than encoding all 44 dimensions in a single *k*-d tree, which would degrade retrieval performance due to the curse of dimensionality, we partition the features by hand (left/right) and type, yielding 8 independent *k*-d trees: 10 dimensions for finger bending angles (Θ), 5 for fingertip touch (*P*), 4 for hand orientation (*Q*), and 3 for hand movement (*A*), each for the left and right hand separately. This decomposition not only improves retrieval speed but also enables independent similarity assessment for each feature type, which is essential for the labeling mechanism described below.

**Figure 4:**
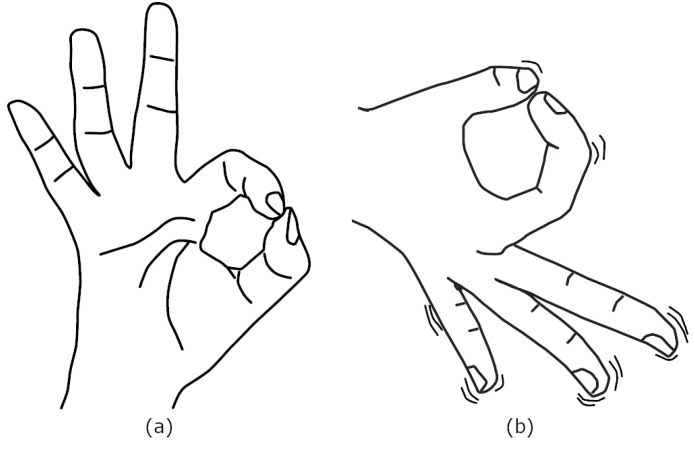
Example of two signs sharing the same hand shape but differing in other features, demonstrating the need for feature labeling. (**a**) “OK” in sign language. (**b**) “Money” in sign language.

Different sign language words may share certain gesture features while differing in others. For example, as shown in Figure 4, “OK” and “money” share the same hand shape but differ in hand orientation and movement. To avoid redundant data entries that would slow retrieval, when a new gesture is added to the dataset, we perform a similarity search against existing entries and assign labels based on the results. Each sign language vocabulary entry is assigned 8 labels, one per feature type, representing its characteristic movement sequence in each *k*-d tree.

**Figure 5:**
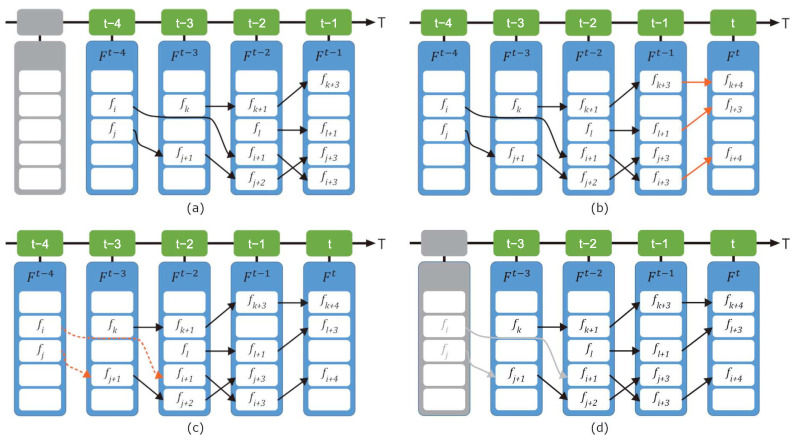
Construction and update of the OLNG with *T* = 4 time intervals and *K* = 5 nodes per time point, illustrating how temporal continuity is maintained through edge connections across consecutive frames. (**a**) OLNG at time t−1. (**b**) At time *t*, new feature data arrives and *k*-NN retrieves *K* similar nodes; edges are created based on temporal continuity. (**c**) The oldest nodes and edges at t−4 are removed. (**d**) Updated OLNG spanning t−3 to *t*. Black arrows denote existing edges, orange arrows the edges newly created at time *t*, dashed orange arrows the edges about to be removed, and grayed-out elements those already removed.

When building labels, we utilize the *k*-NN algorithm to search for the *K* most similar data points in each *k*-d tree for frame *t* of the input gesture. The *k*-NN distances serve as the node costs in the OLNG, which maintains a sliding window of *T* time intervals and *K* candidate paths to track temporal continuity (see Figure 5). The *K* path costs are denoted as $C^{t}=\left\{c_{1}^{t},\ldots ,c_{K}^{t}\right\}$. To convert distances into similarity weights, we normalize $C^{t}$ using Equation (1), yielding path weights $W^{t}=\left\{w_{1}^{t},\ldots ,w_{K}^{t}\right\}$ where higher weights indicate closer matches:

(1) $$w_{i}^{t}=\frac{\max\left(C^{t}\right)-c_{i}^{t}}{\sum_{j=1}^{K}\left(\max\left(C^{t}\right)-c_{j}^{t}\right)}.$$

To assess motion continuity along each OLNG path, we define an energy function that measures the weighted difference between consecutive nodes. Let $f^{t}$ denote the input feature frame at time *t*. The OLNG retrieves the two most recent rows of candidate nodes, $F^{t}=\left\{f_{1}^{t},\ldots ,f_{K}^{t}\right\}$ and $F^{t-1}=\left\{f_{1}^{t-1},\ldots ,f_{K}^{t-1}\right\}$, and computes the path energy between $f^{t}$ and these nodes as:

(2) $$E\left(f^{t}\right)=\sum_{i=1}^{K}w_{i}^{t}\cdot \sqrt{|f^{t}-f_{i}^{t-1}|}.$$

A lower energy value indicates smoother transitions between consecutive frames, meaning the path exhibits greater motion continuity and thus a better match to the input gesture. For each of the 8 feature types, the path with minimum energy identifies the most similar gesture sequence in the dataset. The resulting 8 similarity scores are used to assign labels to the gesture. Each labeled vocabulary *s* is thus represented by a label set $\Gamma_{s}=\left(\gamma_{\Theta_{l}},\gamma_{P_{l}},\gamma_{Q_{l}},\gamma_{A_{l}},\gamma_{\Theta_{r}},\gamma_{P_{r}},\gamma_{Q_{r}},\gamma_{A_{r}}\right)$.

### 4.3. Recognition

**Figure 6:**
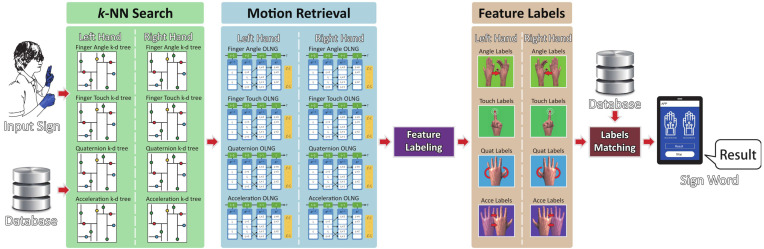
Overview of the recognition pipeline. For each input frame, the *k*-NN algorithm retrieves *K* nearest neighbors from each *k*-d tree, OLNG paths are updated, and the energy function evaluates motion continuity for each path. The vocabulary with the minimum total energy across all feature labels is selected as the recognition result.

After completing the dataset and labels, we use the same preprocessing method as described in Section 4.2 to encode the labeled gesture features using the *k*-d tree algorithm. OLNG is used as the recognition algorithm to calculate the continuity of the feature sequences and the feature labels with the highest similarity. Finally, the sign language vocabulary is selected based on these feature labels. The gesture recognition process is illustrated in Figure 6.

When performing sign language recognition with sign language gloves, given the feature information $\left(\Theta_{l}^{t},P_{l}^{t},Q_{l}^{t},A_{l}^{t},\Theta_{r}^{t},P_{r}^{t},Q_{r}^{t},A_{r}^{t}\right)$ input at time *t*, the *k*-NN algorithm retrieves *K* most similar data from *k*-d tree, denoted as $F^{t}=\left\{f_{1}^{t},\ldots ,f_{K}^{t}\right\}$. The OLNG is utilized to construct the path structure and obtain the path cost, $C^{t}=\left\{c_{1}^{t},\ldots ,c_{K}^{t}\right\}$, and the corresponding path weight, $W^{t}=\left\{w_{1}^{t},\ldots ,w_{K}^{t}\right\}$.

### 4.4. Optimization

After the OLNG algorithm processes the input up to time *t*, it produces *K* best gesture sequence paths and their corresponding path weights $W^{t}$. The labels that these paths belong to are then identified using $F^{t}$, yielding 8 sets of gesture feature labels. The final vocabulary is selected by minimizing the total energy across all feature labels:

(3) $$\begin{array}{cc} s^{*} & =\arg\min_{s}[E\left(f_{\Theta_{l}}^{t}\right)+E\left(f_{P_{l}}^{t}\right)+E\left(f_{Q_{l}}^{t}\right)+E\left(f_{A_{l}}^{t}\right)\\ \; & \;\;+E\left(f_{\Theta_{r}}^{t}\right)+E\left(f_{P_{r}}^{t}\right)+E\left(f_{Q_{r}}^{t}\right)+E\left(f_{A_{r}}^{t}\right)]. \end{array}$$

In Equation (3), $E(f^{t})$ denotes the energy of a particular feature’s OLNG paths during recognition, computed using the same energy function defined in Equation (2), where at recognition time the OLNG nodes at time *t* and t−1 are $F^{t}=\left\{f_{1}^{t},\ldots ,f_{K}^{t}\right\}$ and $F^{t-1}=\left\{f_{1}^{t-1},\ldots ,f_{K}^{t-1}\right\}$.

As in the preprocessing stage, lower energy indicates greater motion continuity, and the vocabulary whose paths yield the minimum total energy across all 8 features is selected as the best match.

During continuous signing, transitional movements occur between consecutive words, making it necessary to distinguish meaningful gestures from transitions. To address this, we compute the average gesture duration τ from the dynamic vocabulary in the dataset and use it as a fixed-length recognition window. Within each window of duration τ, OLNG retrieval and energy computation are performed at every time point *t*. At the end of each window, the energy values for each candidate vocabulary are summed, and candidates with insufficient occurrence frequency are discarded. The vocabulary with the minimum cumulative energy is selected as the final result. This windowed approach effectively filters out transitional movements, which tend to appear infrequently and are thus discarded, reducing misjudgment from non-sign gestures.

## 5. Experiments

We evaluate the proposed system on TSL. Using the TSL Online Dictionary [23], we selected commonly used vocabulary via hand shape search to build a gesture dataset. A total of 300 sign language gestures were recorded by a single participant using our sign language gloves in an indoor laboratory environment, comprising 201 dynamic gestures and 99 static gestures. Each gesture was recorded once and stored directly in the dataset, consistent with the single-sample retrieval design of our OLNG-based approach. Feature labels were established during dataset construction as described in Section 4.2. Storing a single reference sequence per gesture class is a deliberate design choice for efficient vocabulary construction. In this study, the reference vocabulary was recorded once by a single participant, and gestures performed by five additional testers were matched against this common reference; the evaluation therefore provides preliminary cross-participant evidence. However, the limited number and diversity of participants remain a limitation of the current evaluation, and we do not claim broad user-independent generalization.

Five testers (university students, aged 20–25) with no prior sign language experience participated in the evaluation. After receiving instruction on the target gestures, they performed the recognition tasks across all three sign language systems evaluated in this work. All gestures were captured at a 30 Hz sampling rate, and each reference dataset stored a single recorded sequence per gesture class: 26 ASL alphabets, 7 Japanese Sign Language (JSL) sentences, and 300 TSL words. Here, “single-sample” refers to one stored reference sequence per class, not to the number of evaluation trials. In total, the evaluation comprised 650 ASL trials (5 testers × 26 letters × 5 repetitions), 140 JSL trials (5 testers × 7 sentences × 4 repetitions), and 100 TSL trials (5 testers × 20 randomly selected words).

### 5.1. User Evaluation

In the word recognition experiment, each tester randomly selected 20 words from the 300-word dataset. The testers wore the sign language gloves and performed the corresponding gestures, and correctly recognized words were recorded. Individual accuracy was calculated as the ratio of correctly recognized words to the total number of tested words. The average accuracy across all five testers reached 92%, as shown in Table 1.

To contextualize the performance of our proposed system, Table 2 compares it with recent sensor-based sign language recognition studies. These representative studies were selected based on two criteria: (1) use of sign language gloves with flex/IMU sensors and (2) recognition of ASL or JSL gesture sets comparable to our dataset. The comparison highlights sensor configurations, algorithmic approaches ranging from classical machine learning (e.g., SVM, DTW) to deep learning (LSTM), and recognition accuracy under different dataset complexities.

It is important to note that direct comparison across studies is inherently limited, as each system was evaluated on different datasets with varying vocabulary sizes, gesture complexities, and evaluation protocols. For instance, ASL alphabet recognition (26 letters) is a simpler task than recognizing 300 TSL words that include both static and dynamic gestures. For the JSL evaluation, our system was tested on the same 7 sentences used by Chu et al. [13], though the actual gesture data were independently recorded using our own gloves rather than shared across studies. We therefore present Table 2 as a contextual reference rather than a controlled head-to-head benchmark, and we refrain from claiming general superiority over prior systems. Nevertheless, the comparison provides a useful reference for the relative performance of different sensor and algorithm combinations. Our system achieves 96.15% on ASL alphabets, 92.00% on 300 TSL words, and 99.29% on 7 JSL sentences, demonstrating consistent performance across different sign language systems using a single lightweight pipeline. In particular, the JSL evaluation is the most closely aligned setting in Table 2, since both studies consider the same seven sentence categories; our system obtained 99.29% compared with the 96.90% reported by Chu et al. [13]. However, because the gloves, participants, recorded sequences, and evaluation protocols differ, this numerical difference should be read as a contextual value rather than a controlled demonstration of superiority.

### 5.2. Execution Time Analysis

The system was implemented on a low-power Intel N100 mobile processor (Intel Corporation, Santa Clara, CA, USA) [24] using single-threaded C++17 for recognition. During runtime, the execution time of each stage was measured. Table 3 shows the average computation time required for each stage. This implementation setup reflects the design emphasis on translation efficiency and verifies the suitability of the system for embedded deployment, where real-time responsiveness and low computational overhead are critical.

Because prior sensor-based sign language systems generally do not report per-frame or inference time, a direct runtime comparison with them is not possible. We therefore implemented a DTW baseline as a reference point, since DTW—like our method—is training-free and operates on a single recorded reference per gesture, evaluated on the same Intel N100 processor using identical query sequences within each dataset. In contrast, conventional supervised classifiers such as SVM and LSTM typically rely on multiple labeled samples per gesture class for parameter training, whereas our retrieval pipeline performs no parameter training and stores only a single reference sequence per class.

Table 4 reports the per-frame recognition time at two vocabulary sizes. On the small 26-letter ASL alphabet set, DTW is faster (1.24 ms vs. 2.18 ms), as the indexing and labeling overhead of our pipeline does not yet amortize. As the vocabulary grows to 300 TSL words, DTW must align the query against every stored reference sequence, so its per-frame cost increases roughly linearly (from 1.24 to 14.98 ms), whereas our *k*-d-tree-indexed retrieval with feature labeling scales far more gently (from 2.18 to 6.78 ms), making it about 2.2× faster at 300 words. DTW performs exhaustive elastic alignment against every stored reference sequence, which incurs linear-time matching and requires storing every full reference sequence; our indexed retrieval instead reduces redundant feature entries and maintains a lower, more scalable per-frame cost as the vocabulary grows.

### 5.3. Ablation Study

In this study, we incorporated pressure sensors on the finger pads of the sign language glove to detect tactile interactions between the fingers and other body parts. Additionally, during the construction of the gesture dataset, we introduced a labeling strategy based on the similarity of gesture features. This aimed to reduce data redundancy by grouping gestures with similar features while retaining discriminative labels for fine-grained differences.

**Figure 7:**
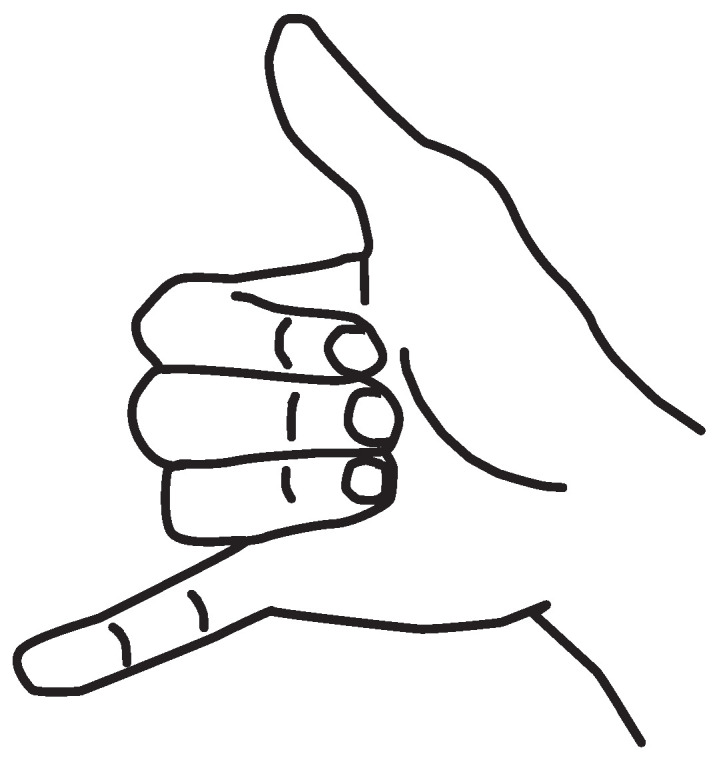
Example of gestures distinguished by touch. Both “telephone” and “salt” use similar hand shapes near the face, but “salt” involves the little finger touching the corner of the mouth. The touch sensor enables the system to differentiate these otherwise similar gestures.

For example, the sign language gestures for “phone ” and “salt” share an identical hand shape, as shown in Figure 7, and are both performed near the cheek. The key difference lies in whether the little finger touches the corner of the mouth. In our TSL dataset, these two gestures are assigned the same labels for finger bending, orientation, and movement features, but are differentiated using distinct touch feature labels. This design allows the recognition system to consider finger contact as a critical cue for distinguishing between otherwise similar gestures.

To assess the contribution of these two design choices, including (1) touch sensors and (2) feature-based labeling, we conducted an ablation study by removing each component independently and measuring the impact on system performance.

Table 5 summarizes the overall accuracy of each configuration. To further analyze the effects of touch sensing and feature labeling, we report per-letter confusion matrices on the ASL alphabet subset. This subset provides a compact set of gestures with several visually similar hand shapes, making it suitable for component-level error analysis.

**Figure 8:**
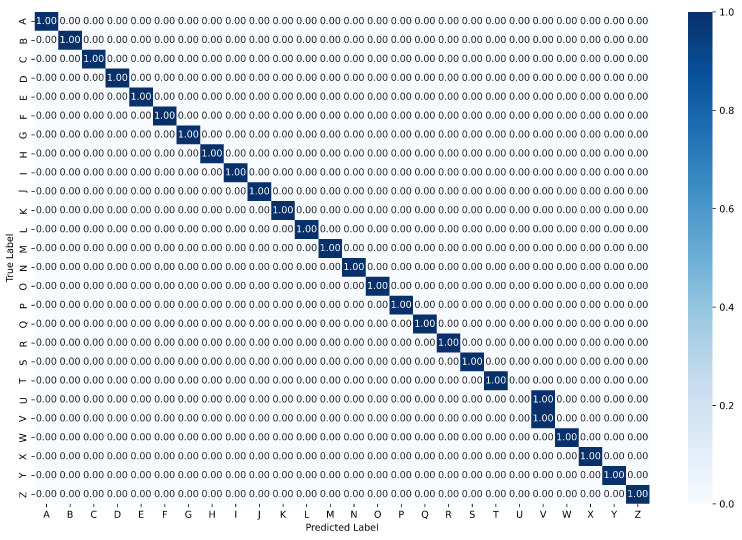
Confusion matrix of the full system on the 26 ASL alphabets. The system fails only on U, which is misclassified as V due to their nearly identical flex-sensor profiles.

**Figure 9:**
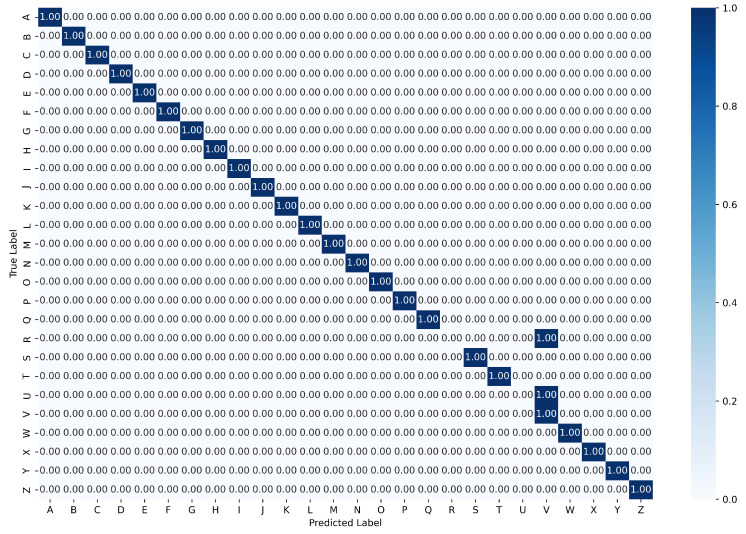
Confusion matrix without touch sensors. In addition to the U–V confusion shared by all configurations, R is misclassified as V, since fingertip contact is the most discriminative cue distinguishing R from V in our glove configuration.

**Figure 10:**
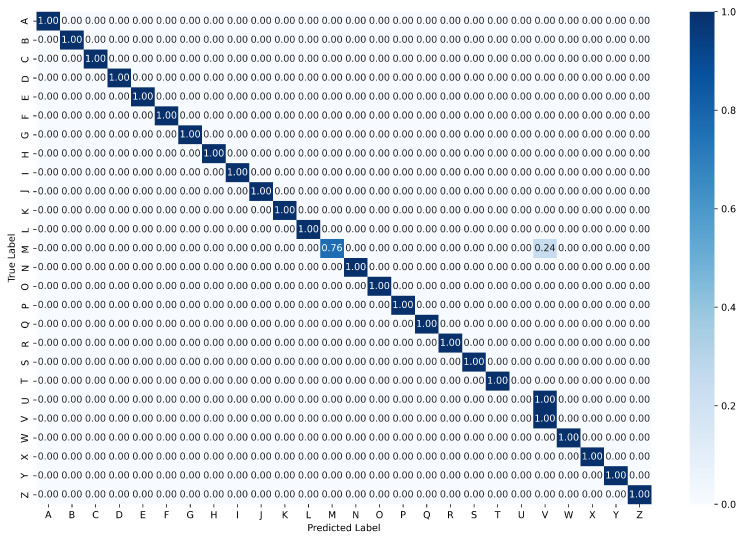
Confusion matrix without feature labeling. Accuracy drops only slightly (M is partially confused with V), but recognition time inflates from 6.78 ms to 161.49 ms per frame due to redundant dataset entries.

As shown in Figure 8, Figure 9 and Figure 10, U is consistently misclassified as V across all configurations. Both letters involve the index and middle fingers being extended, and their main difference lies in the gap between the two fingers. Since the flex sensors primarily measure finger bending and are less sensitive to lateral finger spacing, this confusion reflects a sensing limitation of the current glove design rather than a failure of the retrieval algorithm.

Comparing Figure 8 and Figure 9, removing the touch sensors causes R to be misclassified as V. In our glove configuration, the most discriminative cue between R and V is the fingertip contact pattern caused by crossing the index and middle fingers. Without touch sensing, this cue is no longer available, making R highly similar to V in the remaining flex and orientation features. This result supports the role of fingertip pressure sensing in disambiguating contact-based gestures, and is consistent with the phone/salt example shown earlier in this section.

Comparing Figure 8 and Figure 10, removing feature labeling causes only a small accuracy degradation, but substantially increases recognition time from 6.78 ms to 161.49 ms per frame. This slowdown arises from the size of the per-feature *k*-d trees, which index the individual gesture frames. Without labeling, every frame of all 300 vocabulary entries is indexed, so the eight trees hold 6499, 11, 6687, and 6039 nodes for the left-hand bending, touch, orientation, and movement features, and 6640, 17, 6652, and 6350 nodes for the corresponding right-hand features (about 38,900 nodes in total); this enlarges the candidate space and increases both the nearest-neighbor retrieval cost and the number of downstream OLNG path updates at each frame. With labeling, redundant frames belonging to entries that share the same feature sequence are merged, shrinking the trees to 608, 10, 1440, and 1206 nodes for the left hand and 1086, 14, 2791, and 2463 nodes for the right hand (about 9600 nodes in total); the right hand retains more nodes because many TSL signs are performed one-handed with the right hand. This roughly four-fold reduction in indexed frames—and, above all, the correspondingly smaller number of distinct candidate labels that the label-similarity and OLNG stages must evaluate at each frame—lowers the per-frame cost by more than an order of magnitude. Therefore, feature labeling should be interpreted primarily as an indexing strategy for efficiency, rather than a mechanism for raw accuracy. By reducing redundant feature entries, it enables the retrieval pipeline to maintain real-time performance while preserving comparable recognition accuracy.

## 6. Conclusions

This paper presented a real-time sign language interpretation system that integrates customized sensor gloves with a lightweight motion retrieval algorithm. The system achieved an average recognition accuracy of 92% on a 300-word Taiwanese Sign Language dataset, together with recognition accuracies of 96.15% on the ASL alphabet set and 99.29% on the JSL sentence set. Three practical advantages distinguish our approach: (1) portability—the glove uses BLE and the recognition runs in 6.78 ms on a mobile processor, enabling embedded deployment; (2) low data requirements—unlike deep learning methods that need extensive training data, our OLNG-based retrieval requires only a single recorded sample per gesture; and (3) extensibility—the feature-labeling mechanism allows new vocabulary to be added without retraining.

**Figure 11:**
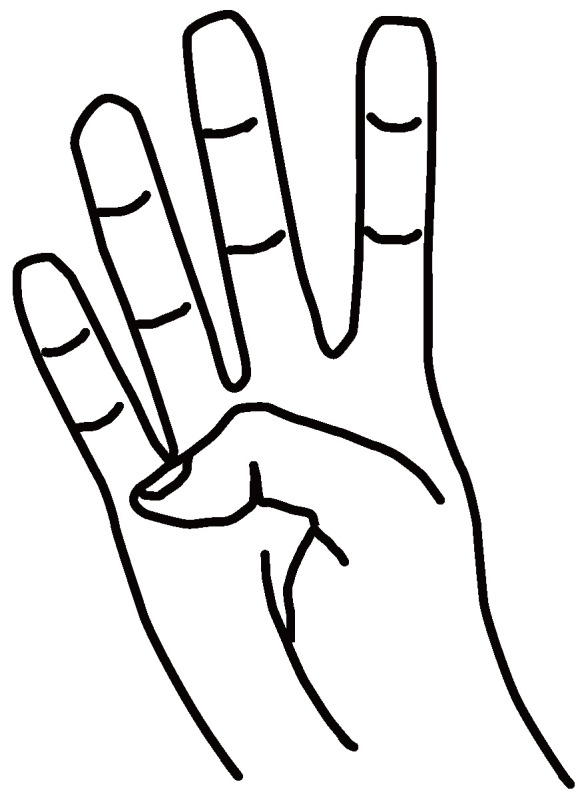
Hand shapes for “please” and “yes.” Both share similar hand shapes but differ in hand position relative to the body and head movement, illustrating a current limitation of the glove-only approach.

**Figure 12:**
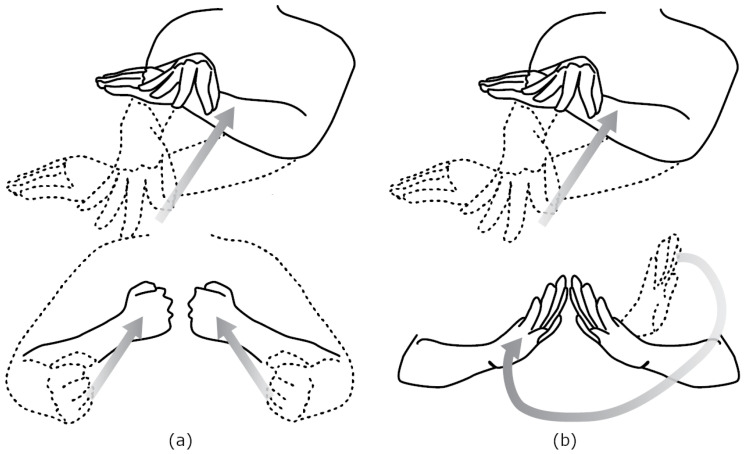
Compound words in sign language. (**a**) Hand shape for “learn.” (**b**) Hand shape for “school.” The arrows indicate the direction of hand movement. Both words start with the gesture “course,” followed by “practice” and “house,” respectively.

Despite these results, several limitations remain:

- Hand position and head movements. The current glove captures hand shape, orientation, movement, and touch, but cannot determine the hand’s absolute position relative to the body. For example, “please” and “yes” share similar hand shapes (Figure 11) but differ in hand position and head movement, leading to recognition errors. Received signal strength indicator (RSSI)-based distance estimation could address this without additional hardware, though receiver placement and signal stability remain open challenges.
- Compound word recognition. Sign language vocabulary can consist of multiple consecutive gestures. For example, both “learn” and “school” (Figure 12, images from [25]) begin with the gesture “course,” followed by “practice” and “house,” respectively. The current system treats each gesture independently, which leads to semantic errors for compound words. Incorporating grammar-level judgment to fuse consecutive gestures into compound vocabulary is an important direction for future work.
- User generalization and vocabulary scaling. The reported results were obtained with a single-participant reference evaluated by five additional testers, providing only preliminary cross-participant evidence. Because signing kinematics and style vary across individuals, a reference recorded by a single participant may not fully generalize to other users. The limited diversity of participants is therefore a limitation, and we do not claim broad user-independent generalization. Building larger multi-participant reference sets and evaluating across more diverse users is an important direction for future work. Separately, although the single-reference-per-class design enables rapid vocabulary expansion, its recognition accuracy and latency have not yet been stress-tested on vocabularies substantially larger than the 300 words evaluated here; characterizing how retrieval accuracy and runtime scale with vocabulary size is another important direction for future work.

## Figures and Tables

**Table 1:** Recognition results of five testers, each performing 20 randomly selected TSL words (average recognition rate 92%).

	Tester 1	Tester 2	Tester 3	Tester 4	Tester 5	Avg.
Correct	18/20	18/20	19/20	18/20	19/20	92%
Accuracy	90%	90%	95%	90%	95%	

**Table 2:** Comparison with recent sensor-based sign language recognition systems. Our system achieves 96.15% on ASL alphabets, 92.00% on 300 TSL words, and 99.29% on 7 JSL sentences.

Reference	Sensors	Accuracy	Dataset	Method
Lee et al. [9]	IMU, flex, and pressure	98.20%	26 ASL alphabets	SVM
Jani et al. [10]	IMU and flex	96.50%	26 ASL alphabets	DTW and nearest mapping
Chu et al. [13]	IMU, flex, and force-sensing resistor (FSR)	96.90%	7 JSL sentences	SVM
Zhang et al. [14]	IMU and flex	98.63%	26 ASL alphabets	Customized LSTM
Ours	IMU, flex, and pressure	96.15% 92.00% 99.29%	26 ASL alphabets 300 TSL words 7 JSL sentences	*k*-NN and OLNG

**Table 3:** Computation times for each stage of the system and the overall recognition result output time.

	Stage	Time
Single Feature	*k*-NN Search	0.04 ms
	OLNG Update	0.05 ms
	Label Similarity	0.75 ms
Overall (All Features)		6.78 ms

**Table 4:** Per-frame recognition time of our method and a DTW baseline on the Intel N100, at two vocabulary sizes.

Method	ASL (26 Letters)	TSL (300 Words)
DTW baseline	1.24 ms	14.98 ms
Ours (*k*-NN and OLNG)	2.18 ms	6.78 ms

**Table 5:** Ablation study on the effects of touch sensors and feature labeling in ASL alphabets.

Method	Accuracy
Full System (with Touch & Labels)	96.15%
Without Touch Sensors	92.31%
Without Feature Labeling	95.23%

## Data Availability

The raw sensor recordings of the 26 ASL alphabet gestures used in the ablation study are included as Supplementary Material. The complete TSL and JSL word and sentence recordings underlying the reported recognition rates are available from the corresponding author on request.

## Supplementary Materials

The following supporting information can be downloaded at https://www.mdpi.com/article/10.3390/s26154884/s1. Dataset S1: Raw sensor recordings of the 26 ASL alphabet gestures used in the ablation study, including a README that documents the recording setup and the sensor-feature column layout.

## Abbreviations

The following abbreviations are used in this manuscript:

TSL	Taiwanese Sign Language
ASL	American Sign Language
JSL	Japanese Sign Language
IMU	Inertial Measurement Unit
ADC	Analog-to-Digital Converter
I2C	Inter-Integrated Circuit
FSR	Force Sensing Resistor
GPU	Graphics Processing Unit
MCU	Microcontroller Unit
BLE	Bluetooth Low Energy
OLNG	Online Lazy Neighborhood Graph
*k*-NN	*k*-Nearest Neighbors
*k*-d tree	*k*-dimensional tree
SVM	Support Vector Machine
DTW	Dynamic Time Warping
LSTM	Long Short-Term Memory
CNN	Convolutional Neural Network
DNN	Deep Neural Network
HMM	Hidden Markov Model
RSSI	Received Signal Strength Indicator
